# Fully Screen-Printed Pressure Sensing Insole—From Proof of Concept to Scalable Manufacturing

**DOI:** 10.3390/s26051456

**Published:** 2026-02-26

**Authors:** Piotr Walter, Andrzej Pepłowski, Filip Budny, Sandra Lepak-Kuc, Jerzy Szałapak, Tomasz Raczyński, Mateusz Korona, Zeeshan Zulfiqar, Andrzej Kotela, Małgorzata Jakubowska

**Affiliations:** 1 Printed Electronics, Textronics & Assembly Lab, Centre for Advanced Materials and Technologies CEZAMAT, Warsaw University of Technology, 19 Poleczki, 02-822 Warsaw, Poland; andrzej.peplowski@pw.edu.pl (A.P.); tomasz.raczynski.dokt@pw.edu.pl (T.R.);; 2 Institute of Mechanics and Printing, Faculty of Mechanical and Industrial Technology, Warsaw University of Technology, 85 Narbutta, 02-524 Warsaw, Poland; 3 Clinic of Rehabilitation and Orthopedics, Department of Rehabilitation and Physiotherapy, Medical University of Lublin, Jaczewskiego 8, 20-090 Lublin, Poland; 4 College of Medicine, Gulf Medical University, Ajman P.O. Box 4184, United Arab Emirates; 5 Faculty of Medicine, Collegium Medicum, Cardinal Stefan Wyszynski University in Warsaw, Wóycickiego 1/3, 01-938 Warsaw, Poland

**Keywords:** wearable gait monitoring, plantar pressure sensing, screen-printed sensors, printed electronics, graphene-based nanocomposites, pressure sensing insole

## Abstract

**Highlights:**

**What are the main findings?**

- Influence of composite formulation, screen mesh density, electrode geometry, lamination, and screen-printing parameters on the resistive response under pressure.
- Scalable fabrication method for flexible pressure-sensing insole based on contact resistance between graphene nanocomposite layer and silver electrode.

**What are the implications of the main findings?**

- Practical process and design guidelines for manufacturing printed pressure sensor insoles for wearable gait monitoring and biofeedback applications.
- Feasibility demonstration of a fully screen-printed multilayer insole architecture compatible with compact readout electronics and everyday footwear.

**Abstract:**

Continuous plantar-pressure monitoring is important for objective gait analysis and early detection of abnormal loading; however, many existing solutions remain laboratory-bound (force plates and instrumented walkways) or rely on costly in-shoe multilayer sensor arrays. Here, we developed and optimized a fully screen-printed pressure-sensing insole based on carbon–polymer nanocomposite layers, with an emphasis on manufacturability and process control to bridge the gap between proof-of-concept force-sensitive resistor (FSR)-based insoles and scalable printed-electronics manufacturing workflows. Composite pastes containing carbon fillers (graphene nanoplatelets, carbon black, and graphite) were formulated to improve sensor repeatability and sensitivity. Sensors were characterized under compression loads from 100 N to 1300 N, showing a sensitivity of 10.5 ± 2.8 Ω per 100 N and a sheet-to-sheet coefficient of variation of 22.1% in resistance response. The effects of paste composition, screen mesh density, electrode layout, and lamination on sensitivity and repeatability were systematically evaluated. In addition, correlation analysis of resistance values from integrated quality-control meanders proved useful for monitoring screen-printing process stability. The final insole integrates printed carbon sensing pads and contacts, a dielectric spacer, and an adhesive layer in a thin, flexible format suitable for integration with wearable electronics. In practical static-load tests, repeated manual placement of weights yielded coefficients of variation as low as 4% at 500 g and a detection limit of ~0.1 N, comparable to a very light finger touch. These results demonstrate that low-cost screen-printed electronics can provide robust pressure sensing for wearable plantar-pressure monitoring.

## 1. Introduction

Gait is a key indicator of functional mobility, and impairments in walking ability are a major target for rehabilitation. In recent years, providing patients with continuous gait monitoring and real-time feedback has emerged as a promising strategy to enhance rehabilitation outcomes [1]. Advances in telemedicine and wearable technology now enable “telerehabilitation” systems that can remotely measure gait parameters and deliver instant feedback to patients in their home environment [2]. This approach gained particular momentum during the COVID-19 pandemic, when remote gait training proved crucial for continuity of care while in-person services were limited [3]. By leveraging telecommunication and sensors, telerehabilitation offers an accessible means to guide therapy and maintain patient engagement outside the clinic.

Wearable sensor platforms for gait analysis have rapidly evolved, enabling objective assessment of walking patterns in real-world settings [4,5]. In particular, instrumented insoles and pressure-sensing mats can capture plantar-pressure data during locomotion, providing rich information on weight distribution and balance. For example, a recent self-powered smart insole system was capable of mapping plantar pressures and recognizing multiple activities using machine learning algorithms [6]. Such foot-pressure devices make it feasible to detect gait events and abnormalities in real time. Martini et al. demonstrated that pressure-sensitive insoles can accurately identify toe-off and heel-strike timings with errors on the order of only 0.04–0.06 s compared to a force-plate gold standard [7]. These wearable gait analysis tools thus enable continuous, quantitative feedback on a patient’s walking performance in daily life.

However, many traditional gait analysis systems remain limited to specialized laboratory settings. Stationary force plates and instrumented walkways, while considered the gold standard for measuring ground reaction forces, are impractical for routine monitoring due to their high cost and lack of portability [8]. Moreover, when patients know they must step on a force plate, they may alter their stride to “target” the plate, leading to atypical gait patterns [9]. In-shoe plantar-pressure systems have improved mobility, but current commercial solutions (e.g., multilayer sensor insoles with tethered electronics) [6] tend to be expensive or cumbersome, posing adoption barriers for widespread daily use. These challenges have driven researchers to explore a range of alternative plantar-pressure and gait-monitoring technologies. A variety of comparative approaches can be found in the literature, each with distinct merits and limitations. For example, Ma and Hu developed a gait analysis setup combining thermal imaging with in-shoe pressure sensors, but the need for a high-speed thermal camera made the system prohibitively costly [10]. Kwon et al. introduced a soft wearable robotic ankle–foot orthosis with built-in pressure sensors and inertial measurement units, capable of providing real-time gait feedback for rehabilitation [11]. While technologically impressive, the added weight and actuation can interfere with the user’s natural movement during everyday walking. Collectively, these examples illustrate innovative approaches to plantar-pressure and gait measurement, while underscoring practical trade-offs in cost, portability, and the degree of intrusion into natural motion.

Meanwhile, recent research efforts have focused on developing novel pressure sensors to improve the comfort and fidelity of wearable gait devices. Printing techniques are opening opportunities to create thin, flexible pressure sensor arrays that can be seamlessly integrated into insoles. For example, Verma et al. reported a 3D-printed piezoresistive sensor using carbon composite inks, which exhibited a stable and repeatable response over a broad pressure range [12]. Likewise, graphene-infused elastomeric composites have yielded flexible pressure sensors with ultra-high sensitivity to small pressure changes [13]. These printed sensors are lightweight, conformable, and cost-efficient, making them ideal for wearable applications. However, to date, most gait-monitoring insoles have relied on discrete, off-the-shelf sensors, and there is a need to translate recent advances in printed sensing technology into practical rehabilitation tools.

In recent years, substantial research has investigated the use of off-the-shelf force-sensitive resistors (FSRs) in instrumented insoles [14,15,16,17]. These systems typically rely on manually attaching sensors at predefined locations to capture plantar pressure [14,15,16,17,18]. Although this approach can be effective in laboratory gait-analysis studies, it has clear limitations for real-world deployment: sensor placement is difficult to reproduce, the assembly process is not scalable, and the bulk of wiring and interconnects makes such systems impractical for everyday use. Advancing the field therefore requires moving beyond proof-of-concept prototypes toward fully printed, integrated insole systems. Bridging this gap is non-trivial. A fully functional insole requires not only development of an appropriate force-sensitive composite and tighter process control, but also re-evaluation of manufacturing methods, as the large-area format strongly influences screen-printing and lamination processes [19]. Moreover, the electrical layout must be adapted to the constraints of printed electronics, while maintaining scalability and repeatability of the overall manufacturing workflow.

In this work, we present a fully screen-printed, low-cost plantar-pressure sensing insole that bridges the gap between lab-scale force-sensitive resistor (FSR)-based prototypes and scalable printed-electronics manufacturing. The study focuses on manufacturing steps and process control, highlighting the factors required to achieve repeatable and manufacturable systems. The novelty of the proposed approach lies in the use of screen printing to fabricate the entire sensing insole—including pressure sensors, conductive interconnects, and the laminated multilayer stack—within a unified, scalable process. Fourteen carbon–polymer ink formulations (based on graphene, carbon black, and graphite) were screened, and printing parameters were optimized to improve sensitivity and repeatability. The resulting thin, flexible insole integrates printed sensing elements, conductive leads, and dielectric spacer layers. To demonstrate end-to-end functionality, the printed insole was integrated with a compact wireless readout module to visualize plantar-pressure maps in real time. Overall, the results indicate that fully printed, low-cost electronics can deliver robust and repeatable pressure sensing for wearable gait-monitoring insoles.

## 2. Materials and Methods

### 2.1. Materials and Reagents

Poly(methyl methacrylate) (PMMA; average molecular weight 3.5 × 10^5^ u) and the solvents 2-(2-butoxyethoxy)ethyl acetate (BEEA) and 2-butoxyethanol (BE) were acquired from Merck Life Science Sp. z o.o. (Poznań, Poland). Laroflex M35 (density 1.24 × 10^3^ kg/m^3^) was purchased from BASF (Ludwigshafen, Germany). Graphene nanoplatelets GNP grade 3 (thickness 8–15 nm, diameter 1–2 μm) were purchased from Cheap Tubes Inc. (Grafton, VT, USA). Graphene nanoplatelets GNP-M25 (mean diameter 25 μm, thickness < 8 nm) were obtained from XG Sciences (Lansing, MI, USA). Carbon black PowCarbon 6315F (CB; particle size 30 nm, surface area 254 m^2^/g) was obtained from Graphene Supermarket (Calverton, NY, USA). Graphite flakes MG 1596 was purchased from Sinograf SA (Toruń, Poland). The insulating layer was printed using DuPont 5018G UV-curable dielectric paste (DuPont, Wilmington, DE, USA). LOCTITE ECI 1010 and LOCTITE DURO-TAK UV 4909 (both Henkel AG & Co. KGaA, Düsseldorf, Germany) were supplied by Grupa MCC Sp. z o.o. (Warsaw, Poland) and used for screen-printing silver electrical traces and the adhesive lamination pattern, respectively. Polyester (PET) foil MYLAR (thickness 125 μm), fabricated by DuPont Teijin Films (Dumfries, UK) was used as the printing substrate.

### 2.2. Preparation of Printing Pastes

Electrically conductive screen-printing pastes for pressure sensors were prepared from carbon functional phase and polymer carrier as summarized in Table 1. Polymer carriers were first obtained by stirring the weighed polymers with their respective solvents (BEEA for PMMA, BEEA and BE for Laroflex) on a magnetic stirrer at 50 °C for 48 h. Conductive carbon composites were then prepared by mixing the carbon functional phase with the corresponding carriers in the weight ratios listed in Table 1, using a planetary mixer (Kakuhunter SK-350T II, Honeystone Ltd., London, UK; 2200 rpm revolution, 70 rpm rotation). The pastes were subsequently homogenized on a three-roll mill (EXAKT 80E; roller gap 5 µm, torque 0.2 N/mm).

### 2.3. Insoles Fabrication

**Figure 1:**
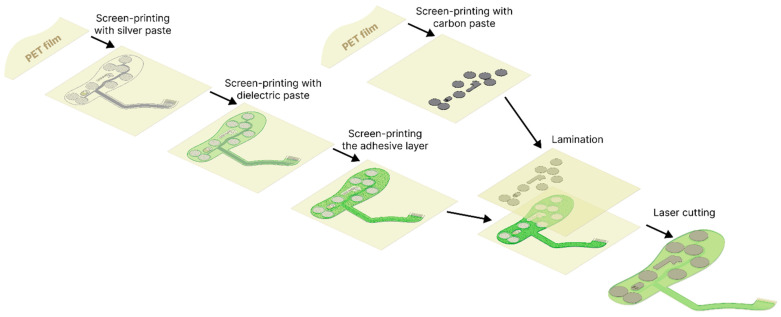
Step-by-step process of insoles fabrication.

For each insole size, four screen patterns were designed and used sequentially to print the silver, dielectric and adhesive layers on the first PET film substrate, and the carbon sensor layer on the second PET film substrate (Figure 1). Both substrates consisted of 125 µm-thick PET film; heat-treated at 130 °C for 40 min to minimize shrinkage during subsequent thermal curing of pastes. Screen printing was carried out on a semi-automatic printer (Aurel C920, AUREL s.p.a., Modigliana, Italy). Printing parameters (squeegee position and deflection, movement speed, screen off-contact) were adjusted for each paste to obtain a continuous layer in a single squeegee pass. The GNP-based sensor pastes and the silver paste were screen-printed and thermally cured at 120 °C for 20 min. On the substrate bearing the silver traces, the dielectric paste was then deposited and UV-cured, acting both as electrical insulation and as a mechanical spacer between the two insole sides. A UV-curable pressure-sensitive adhesive layer was subsequently printed in the lamination areas. Based on alignment markers printed on both PET substrates, registration holes were laser-cut to enable reproducible positioning for lamination. After bonding, the insole outline and the electrical signal leads were laser-cut to their final shape.

**Figure 2:**
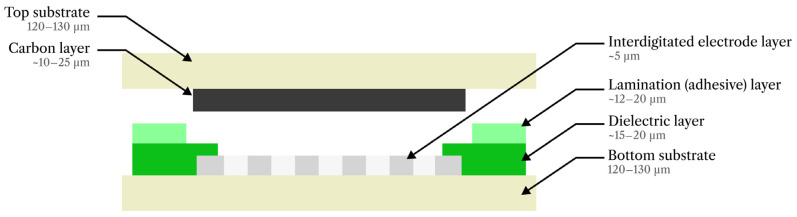
Cross-sectional schematic of the pressure sensor depicting the layer stack and typical thicknesses of the screen-printed layers and PET substrates.

All insoles were fabricated with polyester screens patterned with a UV-curable emulsion (Maroka Polska Sp. z o.o., Katowice, Poland). The screen designs defined the GNP composite sensor layer on one PET substrate and, on the second substrate, the silver contact layer with pads for electrical connection and the overlying dielectric/spacer regions, as illustrated in Figure 2.

### 2.4. Pressure–Resistance Response Measurements

**Figure 3:**
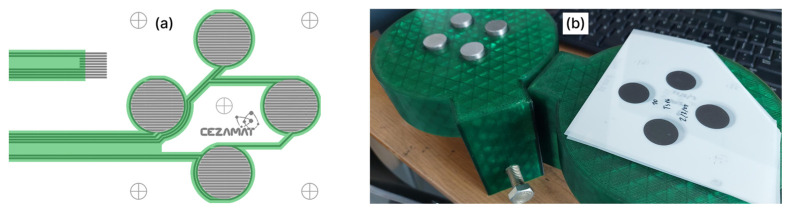
Sensors used for pressure–resistance measurements: (**a**) CAD layout of the four-sensor set; (**b**) custom measurement setup installed in the UTM.

The screen-printed pattern designed for compression measurements consisted of a set of four circular pressure sensors (Figure 3a). Each sensor featured an active area with a diameter of 25 mm, defined by the outline of the dielectric layer (Figure 2). The manufacturing followed the same steps as insoles fabrication (Section 2.3) with the exception that the patterns were not laser-cut and in the initial series of experiments (Section 3.1, Section 3.2, Section 3.3 and Section 3.4), neither the dielectric layer nor the adhesive layer was applied.

To carry out compression tests on the universal testing machine (UTM), the sensors were placed in a custom-made setup in which each sensor was aligned beneath one of 4 steel discs (Figure 3b). The cylinders were pressed with a polymer spacer between the disc and the upper table allowing compensation (through plastic deformation) for minor surface irregularities of the table, steel discs or potential setup skew.

**Figure 4:**
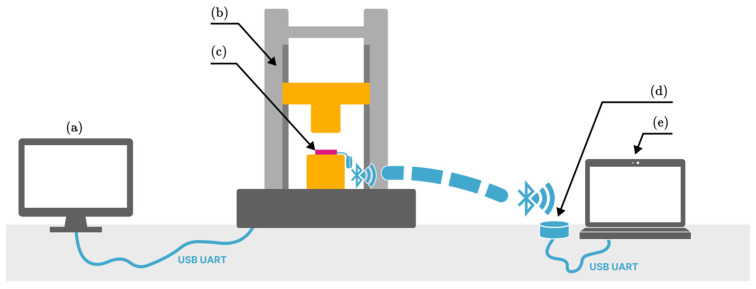
Pressure–resistance response measurements setup: (**a**) Universal testing machine control computer; (**b**) UTM: two-column Cometech QC-560 M2 tensile machine (Taichung City, Taiwan) with 10 kN pressure gauge; (**c**) pressure sensors undergoing compressive loads; (**d**) remote resistance readout module; (**e**) resistance readout module control computer.

Simultaneously with force acquisition by the UTM, the resistance response of the four-sensor set was recorded using a self-developed readout module (Figure 4). Both signals were time-synchronized and subsequently sampled to obtain the resistance–pressure dependence over a force range of 100–1300 N, with increments of 100 N. For each applied load level, resistance values measured for sensors of the same type were grouped and statistically analyzed. Outliers were identified and removed using the box-and-whisker method. The applied compression force was divided by four for data representation, yielding the load acting on an individual sensor. Resistance readout module’s measurement range was limited to 100–5000 Ω in the initial tests (Section 3.1 and Section 3.2), as it was the target working range in the process of preliminary screening. In subsequent tests, a setup with a switchable resistor and thus a higher measurement range was used to comprehensively characterize the influence of the investigated parameters on sensors fabricated using the selected composition.

## 3. Results

### 3.1. Screening of Composite Formulations for Pressure-Sensitive Layers

The functional phase concentration and the carrier system, including the polymer type and its concentration, were jointly investigated due to their coupled influence on composite rheology and—subsequently—on the electrical properties of the printed layers and their sensitivity under load. In total, the screening comprised eleven composite formulations, denoted TS01–TS11, whose compositions are summarized in Table 1.

**Figure 5:**
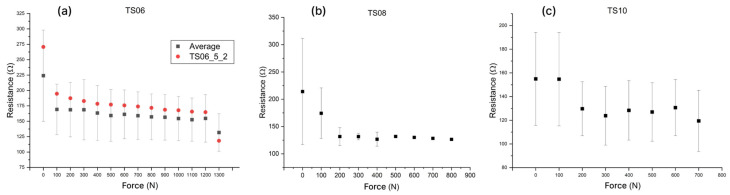
Charts presenting the resistive responses of the tested formulations; (**a**) TS06; response from one sensor is highlighted (**b**) TS08 (**c**) TS10.

All pastes were screen-printed through a 77T mesh screen and subsequently evaluated as a resistive pressure-sensitive layer under increasing normal load. From all the samples, only ones printed with TS06 and TS10 showed a pronounced change in resistance with applied force above 300 N, whereas TS07, TS08, TS09, and TS11 exhibited only minor resistivity variations, indicating low sensitivity and poor suitability as pressure sensors. The resistive responses of TS06, TS08 and TS11 carbon composites are presented in Figure 5. Crucially, the error bars denote the standard deviation of resistance values obtained from multiple sensor sets of the same type and therefore reflect sample-to-sample variability rather than the measurement uncertainty of a single sensor. To demonstrate that pressure could be inferred from the resistance signal, the response of one sensor set is shown (Figure 5a), exhibiting a monotonic resistance–pressure relationship.

Among the tested formulations, TS06 provided the widest usable measurement range and the highest sensitivity, thereby defining its carrier type and carbon phase concentration as the baseline formulation for further refinement.

### 3.2. Effect of Carbon Filler Type and Content on Sensor Response

**Figure 6:**
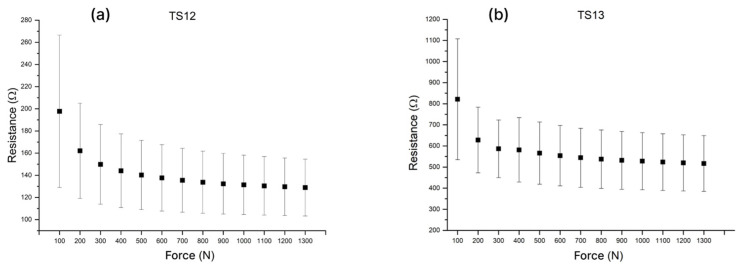
Resistive response of formulations with different carbon fillers: (**a**) GNP of a different size (TS12); (**b**) GNP and CB (TS13).

The second series of experiments was designed to optimize the baseline TS06 formulation by modifying the conductive phase of the composite paste, with the aim of further improving sensor sensitivity and repeatability. With the polymer carrier and the overall conductive-phase loading kept constant, graphene nanoplatelets of a different size (TS12) and a partial substitution of graphene nanoplatelets with carbon black (TS13) were investigated. In addition, a graphite-flake-based paste (TS14) was evaluated, using a higher filler loading to achieve comparable conductivity. Formulations TS12–TS14 (Table 1) were screen-printed using a 77T mesh. The resistive responses obtained for TS12 and TS13 are shown in Figure 6.

Sensors fabricated using the graphite-flake formulation (TS14) exhibited resistance responses exceeding 10 kΩ. Given the susceptibility of high-impedance measurements to electrical noise and interference, and considering the intended application, this resistance level was deemed unsuitable. Because TS14 already required a substantially higher graphite loading (10 wt% vs. 5 wt% in the baseline formulation), further increases in the conductive phase were not considered practical due to printability and film-quality limitations. Therefore, graphite-flake-based pastes were not pursued further in this work.

As shown in Figure 6a, TS12 exhibits a relatively wide spread of resistance at lower loads (≈100–400 N), which narrows as the pressure increases. It corresponds with strong response at lower loads but limited sensitivity in the upper pressure range. TS13 (Figure 6b) similarly shows pronounced variability at very low loads (<100 N), followed by a more repeatable (sample-to-sample) response above 200 N. When compared with TS06, both TS12 and TS13 generally display higher variability of the resistance response between manufactured sensors—both in terms of relative as well as absolute resistance value. One of the main aims of this work is to tackle the challenge of manufacturing repeatability of the resistive pressure-sensitive sensors, and among the tested compositions TS06 remained the most balanced formulation in terms of repeatability and sensitivity across a wide pressure range. Hence, despite promising results obtained with other carbon fillers (graphite flakes and GNPs of different sizes), TS06 was selected as the baseline composition for subsequent experiments.

### 3.3. Influence of Screen Mesh Density on Pressure Sensor Performance

In the third series of tests, the influence of screen mesh density on sensor sensitivity and repeatability was investigated. The TS06 formulation was screen-printed using meshes with four thread counts: 43T, 60T, 77T, and 150T.

**Figure 7:**
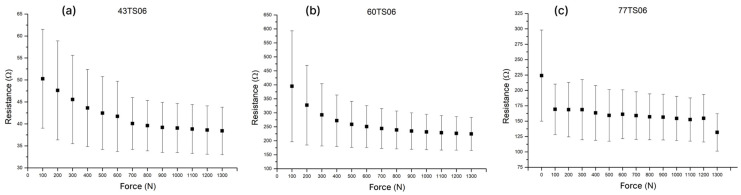
Resistive responses of the TS06 formulation using screens with different mesh counts. (**a**) 43T, (**b**) 60T, (**c**) 77T.

The densest mesh (150T) was characterized by a relatively small mesh opening in relation to the viscosity of the TS06 paste, as well as a significantly lower mesh thickness, which resulted in a substantially thinner printed layer. Consequently, the fabricated sensors exhibited resistance values exceeding the upper limit of the measurement range of the readout module. The remaining mesh thread counts yielded functional sensors, whose performance is presented in Figure 7.

Among tested meshes, it was observed that sensor pads printed with 43 thread count yielded the highest relative resistance change in response to pressure, and the mean value change was the most consistent throughout the measured range. Improved linearity combined with good repeatability led to the selection of the 43T mesh density for all subsequent experiments.

However, a lower thread count is inherently associated with higher mesh thickness and a greater total amount of deposited ink [20]. Consequently, the produced layers exhibit higher conductivity, as shown in Figure 7a, where throughout the pressure range, the average resistance changed by only 12 Ω. To address that, whilst retaining the benefits of the low thread count, it was hypothesized that the problem could be mitigated by reducing the number of fingers in the interdigitated electrode.

### 3.4. Impact of Electrode Layout on Sensitivity and Repeatability

**Figure 8:**
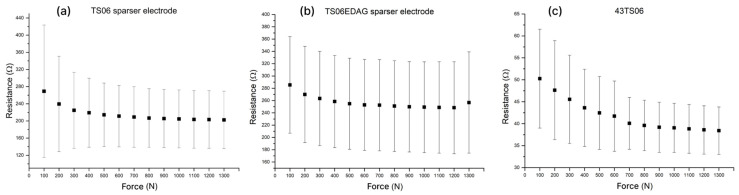
Resistive responses of the TS06 formulation printed using a 43T mesh: (**a**) new sensor design with sparser contacts; (**b**) new sensor design with sparser contacts printed with an alternative silver paste; (**c**) previous electrode design.

To increase the resistance output span and, consequently, the sensor sensitivity, a new electrode layout with sparser fingers in the interdigitated electrode was introduced. The design is based on the premise that reducing the contact area between the silver electrodes and the carbon resistive layer increases effective resistance and concentrates the electric field at the contact points. This higher local field and current density amplify resistance changes under load, improving sensitivity to small pressure variations and supporting more consistent measurements across the pressure range. In addition, a larger spacing between adjacent electrodes reduces the influence of local printing defects in the silver layer, as such defects have a lower relative impact on the effective conduction path length between corresponding interdigitated electrodes. The resistive responses of the new and previous electrode designs were compared (Figure 8a and Figure 8c respectively).

Along with the new electrode layout, an alternative silver printing paste was also evaluated, in which the interdigitated electrodes were printed using EDAG 725A instead of ECI 1010. This paste was not employed in subsequent experiments, as the overall nature of the pressure–resistance relationship remained unchanged. Nevertheless, the data obtained for electrodes printed with ECI 1010 is shown on Figure 8b to demonstrate that the sensitivity of the investigated sensors is governed primarily by the carbon-based resistive layer rather than by the silver electrode material.

As expected, the sensor resistance increased significantly as the reduced number of fingers in the interdigitated electrode yielded fewer contact points with the carbon pad. Consequently, the resistance change within the investigated pressure range increased, improving sensitivity in most cases, although this was less evident in the mean plot due to outliers resulting from sensor-to-sensor variability. At this stage, it was decided to incorporate lamination in the fabrication procedure to eliminate potential errors stemming from manual alignment of the carbon pads over the interdigitated silver electrode or substrate movement during measurements.

### 3.5. Effect of Lamination and Layer Alignment on Sensor Behaviour

**Figure 9:**
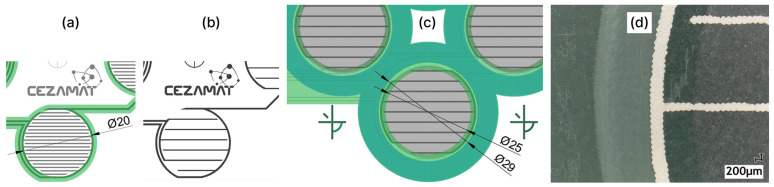
Evolution of the electrode and lamination layer designs: (**a**) initial layout, in which the lamination layer coverage matched the insulating-layer outline; dimensions in mm; (**b**) modified interdigitated electrode with a reduced number of fingers used for tests described in Section 3.4; other dimensions and layers were unchanged at that stage; (**c**) modified design with an enlarged electrode and increased lamination layer openings to avoid overlap with the sensor active area; dimensions in mm; (**d**) microscope imaging of the printed sensor demonstrating dielectric and lamination layers spacing.

To investigate the influence of lamination on the pressure-sensing behaviour of the printed sensors, the insulating layer was printed on all samples, and an additional adhesive layer for lamination was applied to half of them. At this stage, the alignment procedure was refined, and the lamination screen design was modified by increasing the diameter of the openings in the lamination layer to prevent adhesive encroachment into the electrode area, as illustrated in Figure 9c,d.

**Figure 10:**
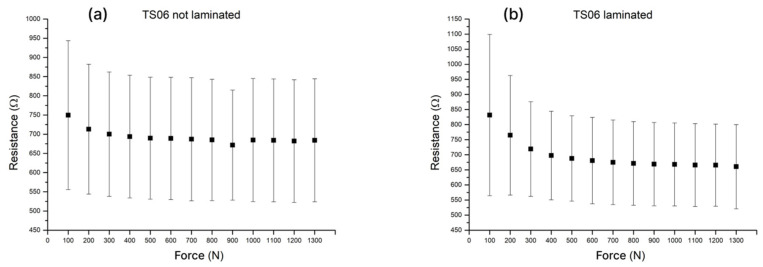
Resistive responses of the TS06 formulation printed using a 43T mesh: (**a**) non-laminated sensors; (**b**) laminated sensors.

The performance of laminated and non-laminated sensors is compared in Figure 10. Owing to the additional layers, which introduce spacing between the silver electrode and the carbon pad, the baseline resistance increased markedly compared to previous tests. The experiments further showed that lamination substantially improved both sensor sensitivity and sensor-to-sensor repeatability. This improvement is attributed to more reliable alignment of the carbon pads over the interdigitated silver electrodes and to suppression of substrate movement during measurements.

Laminated sensors exhibited a sheet-to-sheet coefficient of variation in resistance response of 22.1%. Sensitivity was evaluated from the resistance–force relationship of individual sensors by fitting a linear regression (least-squares method), with the absolute slope taken as the sensor sensitivity. The mean sensitivity across the full analyzed range (100–1300 N) was 10.5 ± 2.8 Ω per 100 N (n = 37). Because the per-sensor force during practical use is expected to be well below 1300 N (i.e., the plantar load is distributed across multiple sensors), the non-linear resistance–force response was also evaluated in two subranges, 100–400 N and 400–1300 N, yielding sensitivities of 54 ± 15 Ω/100 N and 3.49 ± 0.73 Ω/100 N, respectively.

### 3.6. Influence of Screen-Printing Parameters on Resistivity and Repeatability

**Figure 11:**
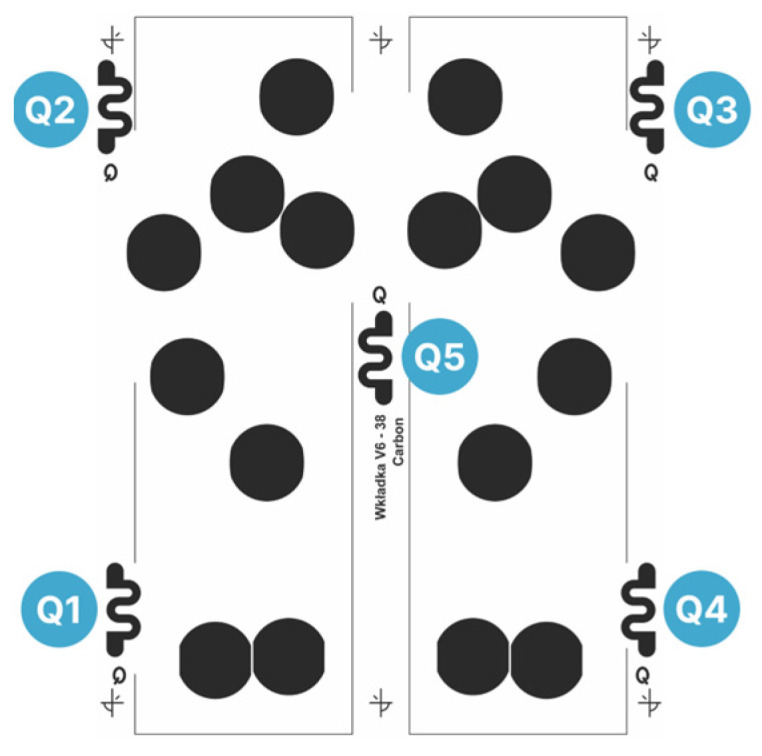
Modified screen layout incorporating five QC meander structures for monitoring layer resistivity and printing uniformity.

To control sheet-to-sheet repeatability—particularly with a view toward scaled manufacturing—quality-control (QC) structures in the form of meander patterns were incorporated into the screen designs of the carbon layer (Figure 11).

It was hypothesized that resistance measured at multiple locations across a printed sheet could serve as a proxy for the uniformity and resistivity of the sensing layer. To evaluate this assumption, the effects of selected screen-printing parameters on layer resistivity and repeatability were investigated. The TS06 composition was screen-printed while systematically varying the screen–substrate distance, squeegee pressure, and squeegee speed.

The screen–substrate distance was adjusted between 4 and 7 mm. This parameter affects the snap-off behaviour (i.e., how cleanly the screen separates from the substrate after the squeegee pass), which in turn influences layer uniformity and edge definition. The print-head position was varied from −0.25 to 1 mm, corresponding to an estimated squeegee force of approximately 6–35 N. Adequate pressure is required to drive the paste through the mesh while avoiding incomplete coverage (at too low pressure) or excessive spreading and overly thick deposits (at too high pressure). The squeegee forward speed was varied from 150 to 300 mm/s. Squeegee speed affects paste transfer through the mesh and levelling on the substrate, thereby influencing layer thickness, coverage, and reproduction of fine features.

In total, 200 samples were printed across the tested parameter combinations, and the resistivity of the QC meanders was measured. Pearson correlation analysis was then used to quantify relationships between the printing parameters, resistivity, and repeatability, enabling identification of conditions that improve layer consistency. The key parameters are summarized in Table 2.

The screen–substrate distance, although a crucial printing parameter, was excluded from the Pearson correlation analysis presented below. When set too low, snap-off is insufficient and print uniformity deteriorates; when set too high, paste transfer becomes incomplete and coverage is reduced. Because print quality degrades on both sides of an intermediate process window, this parameter is not expected to exhibit a linear correlation with resistivity-based quality indicators over a sufficiently broad range. Consistent with this, no meaningful correlation was observed between screen–substrate distance and the resistance of the QC meanders (Q1–Q5). In contrast, print-head position and squeegee speed were varied within a constrained interval within or near the process window, for which approximately linear trends are more plausible and thus suitable for Pearson correlation analysis.

## 4. Discussion

### 4.1. Manufacturing Process Control

The Pearson’s correlation analysis for the quality-control (QC) meanders (Q1–Q5) shows that printing parameters have a measurable impact on both the absolute resistance and its uniformity across the sensor layer.

In particular, forward speed exhibits a very strong negative correlation with meander resistance, but negligible effect on spread metrics. This is highly relevant for manufacturing a pressure-sensitive layer as it indicates that the output resistance of the print can be fine-tuned by adjusting the squeegee speed without compromising process repeatability. The strong correlation observed for Q1, Q2 and Q4, as well as for the average resistance (*p* < 0.02), further suggests that the practical process window may extend beyond the tested range, and that speeds below 150 mm/s and above 300 mm/s could be evaluated in future work. The findings of Potts et al. [21] corroborated the decrease in paste deposition within this speed range, reporting significantly lower film thickness and line width when the squeegee speed was increased from 100 mm/s to 300 mm/s. However, the relationship is not monotonic: at 500 mm/s, higher ink deposition was again observed, resulting in a >20% decrease in line resistance.

Considering the four meanders located in the corners of the layout (Q1–Q4), higher mean meander resistance tended to coincide with higher variability, suggesting that elevated resistivity may arise when printing conditions drift outside the process window and simultaneously increase within-batch inconsistency. To maximize sensitivity to such process deviations, QC markers should be positioned in regions of the screen that are most susceptible to printing artefacts, i.e., toward the periphery of the mesh where distortions are expected to increase as the squeegee approaches the ends of the mesh [20,22]. Consistently, the centrally located QC meander—unlike the corner markers—showed no meaningful correlation with either the sample-mean resistance or the statistical spread metrics, aligning with practical experience that the central part of the print is produced with higher reliability than features located near the pattern edges and the screen frame [19,20,22,23]. Overall, these results confirm that simple QC meander structures provide a sensitive indicator of process stability and can be used to refine printing conditions such that the pressure-sensing insole combines suitable sheet resistance with good repeatability of the sensing behaviour.

**Figure 12:**
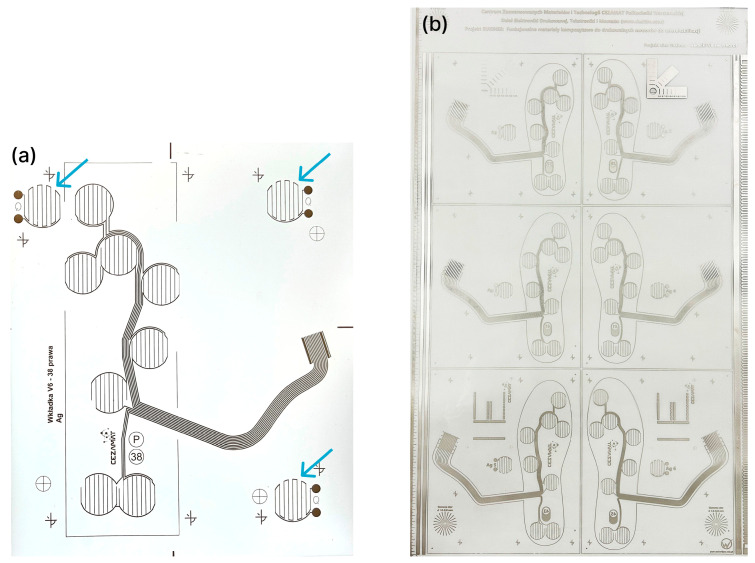
(**a**) Silver-layer layout incorporating three quality-control meanders; (**b**) print from the trial batch produced using a 1200 × 1600 mm screen with 600 × 1000 mm active print area.

Given the utility of this approach for process control, QC meanders were also incorporated into the silver-layer layout (Figure 12a). Notably, the meander resistance also proved useful during scale-up, serving as a reference metric for comparing prints produced using 1200 × 1600 mm screens (Figure 12b).

### 4.2. Applicability for Plantar-Pressure Measurements/Gait Analysis

Crucially, this study focuses on sensor manufacturing, including development of the carbon paste for the pressure-sensitive layer, electrode design, evaluation of sensor-to-sensor repeatability and sensitivity, and process control. While subsequent plantar-pressure studies and clinical evaluation are addressed in a separate study, a brief functional demonstration is included here to confirm that the manufacturing optimizations yielded a working prototype fit for purpose.

**Figure 13:**
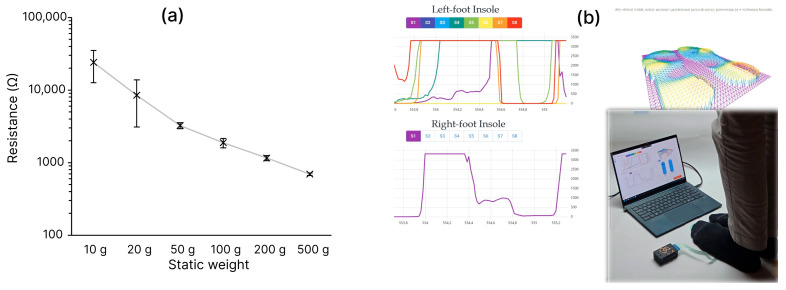
(**a**) Resistance response under manually applied dead weights with repeated removal and reapplication between measurements. (**b**) 3D map of plantar-pressure indicators obtained with the developed insole system (still frame from Supplementary Material Video S1).

For a preliminary assessment of sensing functionality, calibrated dead weights were applied to a single pressure sensor while its resistance was continuously recorded. In this experiment, we assessed within-sensor measurement repeatability under manual repositioning, in contrast to the earlier experiments focused on sensor-to-sensor repeatability. For each load level, the weight was manually removed and reapplied to introduce realistic variability in placement. To accommodate minor surface irregularities and better approximate a compliant contact interface, the load was applied through a compliant rubber-foam spacer (20 mm diameter). The spacer mass (0.25 g) was neglected in the presented data for clarity. The resulting resistance values are shown in Figure 13a. In the unloaded state, the sensor behaved as an open circuit (R > 1 GΩ). The lowest load producing a measurable resistive response was 10 g (≈0.1 N). Across repeated placements, the coefficient of variation ranged from 64% at 20 g to 4% at 500 g. These results indicate a very low detection threshold of the insole, down to approximately 0.1 N, which is below a very light finger touch [24].

To illustrate applicability to plantar-pressure measurements and the potential for posture or gait assessment, we also performed a system-level demonstration in which the manufactured insole, together with a self-developed data-acquisition module and a computer application, provides real-time biofeedback from eight sensors distributed across the insole. The 3D map (Figure 13b) displays the resistance of individual sensors, converted into relative pressure indicators. A full demonstration video is provided in Supplementary Material Video S1.

## 5. Conclusions

This work presented the development and optimization of a screen-printed pressure-sensing insole based on carbon/graphene nanocomposite layers for plantar-pressure measurement and wearable gait monitoring. A series of formulation and process studies was carried out to identify a resistive sensing layer that combines high sensitivity with acceptable repeatability under loads representative of plantar forces. Among the tested composites, an optimized graphene–polymer formulation provided the most balanced response, enabling stable resistance changes over a wide pressure range.

Beyond material selection, we systematically investigated key technological parameters of the screen-printing process and device architecture. The studies demonstrated that screen mesh density, electrode layout, lamination quality and printing parameters (screen–substrate distance, squeegee pressure and speed) all have a pronounced impact on sensor behaviour. Printing through a low-count mesh and a sparse electrode design yielded the highest sensitivity and improved repeatability, while careful control of lamination alignment and the introduction of printed quality-control structures were essential to maintain stable sensor responses.

**Figure 14:**
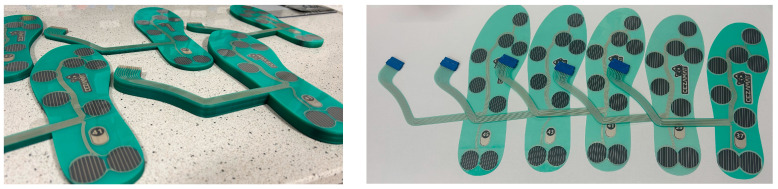
Stacks of fully screen-printed, pressure-sensitive insoles after lamination (**left**); individual units equipped with connectors in sizes 37, 39, 41, 43, and 45 (**right**).

Using optimized materials and processes, fully functional, fully screen-printed pressure-sensing insoles were fabricated on flexible PET substrates (Figure 14). These prototypes integrate printed sensing, contact, dielectric and adhesive layers into a thin, conformable structure that is compatible with wearable gait-monitoring setups. Although the present evaluation focused on quasi-static compression tests, the obtained results indicate that the proposed technology provides a robust basis for scalable fabrication of pressure-sensing insoles.

Clinical validation and gait-derived outcomes are addressed in a companion study, building directly on the manufacturing and sensor-development results reported here. Future work will address dynamic testing, long-term stability and calibration under repeated loading cycles, as well as integration with compact wireless electronics and data-processing algorithms. Overall, the findings of this study provide practical guidelines for the formulation and manufacturing of printed resistive pressure sensors and demonstrate the feasibility of a screen-printed pressure-sensing insole as a candidate platform for wearable plantar-pressure monitoring in both clinical and everyday environments.

## 6. Patents

The work reported in this manuscript is related to the following patent application:“Wkładka czujnikowa do pomiaru rozkładu nacisku na stopę podczas chodu oraz sposób jej wytworzenia” (Sensor insole for measuring plantar-pressure distribution during gait and method of its fabrication), Polish patent application No. P.453191, submitted to the Patent Office of the Republic of Poland (UPRP).

## Figures and Tables

**Table 1:** Summary of tunable screen-printing composites (TS) tested as a resistive pressure-sensitive layer. GNP* regards the GNP-M25 carbon phase, as the other GNP regards the GNP grade 3.

Notation	Functional Phase	Concentration of the Functional Phase	Carrier Polymer	Concentration of the Polymer in the Carrier
TS01	GNP	5%	Laroflex	24%
TS02	GNP	7%	Laroflex	24%
TS03	GNP	5%	Laroflex	40%
TS04	GNP	5%	Laroflex	35%
TS05	GNP	5%	PMMA	16%
TS06	GNP	5%	Laroflex	30%
TS07	GNP	7%	Laroflex	30%
TS08	GNP	5%	PMMA	12%
TS09	GNP	7%	PMMA	12%
TS10	GNP	5%	PMMA	10%
TS11	GNP	7%	PMMA	10%
TS12	GNP*	5%	Laroflex	30%
TS13	GNP+CB	4.5% + 0.5%	Laroflex	30%
TS14	Graphite flakes	10%	Laroflex	30%

**Table 2:** Pearson’s correlation analysis for the quality-control (QC) meanders (Q1–Q5) and selected printing parameters.

Parameter	Print Head Position	Forward Speed	Average Q1–Q5 Resistance	Single Sheet Q1–Q5 SD	Single Sheet Q1–Q5%RSD
**Meander Q1**	−0.54 (*p =* 0.14)	**−0.90 (*p =* 0.00)**	**0.88 (*p =* 0.00)**	−0.04 (*p =* 0.93)	−0.36 (*p =* 0.34)
**Meander Q2**	−0.50 (*p =* 0.17)	**−0.78 (*p =* 0.01)**	**0.85 (*p =* 0.00)**	−0.14 (*p =* 0.73)	−0.35 (*p =* 0.35)
**Meander Q3**	−0.30 (*p =* 0.43)	−0.56 (*p =* 0.12)	**0.66 (*p =* 0.05)**	**−0.67 (*p =* 0.05)**	**−0.79 (*p =* 0.01)**
**Meander Q4**	−0.59 (*p =* 0.10)	**−0.81 (*p =* 0.01)**	**0.96 (*p =* 0.00)**	−0.06 (*p =* 0.88)	−0.37 (*p =* 0.33)
**Meander Q5**	0.10 (*p =* 0.81)	−0.31 (*p =* 0.42)	0.40 (*p =* 0.29)	**0.67 (*p =* 0.05)**	0.55 (*p =* 0.12)
**Single sheet Q1** **–** **Q5 average**	−0.49 (*p =* 0.18)	**−0.90 (*p =* 0.00)**	1.00 (*p =* 1.00)	−0.06 (*p =* 0.87)	−0.36 (*p =* 0.34)
**Single sheet Q1** **–** **Q5 SD**	0.36 (*p =* 0.34)	0.03 (*p =* 0.94)	−0.06 (*p =* 0.87)	1.00 (*p =* 1.00)	0.93 (*p =* 0.00)
**Single sheet Q1** **–** **Q5%RSD**	0.55 (*p =* 0.12)	0.29 (*p =* 0.44)	−0.36 (*p =* 0.34)	0.93 (*p =* 0.00)	1.00 (*p =* 1.00)

Bold values indicate statistically significant correlations (*p* ≤ 0.05).

## Data Availability

The original contributions presented in this study are included in the article/Supplementary Materials. Further inquiries can be directed to the corresponding author.

## Supplementary Materials

The following supporting information can be downloaded at: https://www.mdpi.com/article/10.3390/s26051456/s1, Video S1: Real-time mapping of pressure indicators using the fully screen-printed pressure-sensing insole.

## Abbreviations

The following abbreviations are used in this manuscript:

PMMA	Poly(methyl methacrylate)
BE	2—butoxyethanol
BEEA	2-(2-butoxyethoxy)ethyl acetate
GNP	Graphene nanoplatelets
CB	Carbon black
